# Edible Plants and Their Influence on the Gut Microbiome and Acne

**DOI:** 10.3390/ijms18051070

**Published:** 2017-05-17

**Authors:** Ashley K. Clark, Kelly N. Haas, Raja K. Sivamani

**Affiliations:** 1 School of Medicine, University of California–Davis, Sacramento, CA 95816, USA; akclark@ucdavis.edu; 2 Department of Dermatology, University of California–Davis, Sacramento, CA 95816, USA; kelly.nicole.haas@gmail.com; 3 Department of Biological Sciences, California State University, Sacramento, CA 95819, USA

**Keywords:** acne, gastrointestinal tract, skin, microbiota, botanicals, polyphenols, probiotics, insulin resistance

## Abstract

Acne vulgaris affects most people at some point in their lives. Due to unclear etiology, likely with multiple factors, targeted and low-risk treatments have yet to be developed. In this review, we explore the multiple causes of acne and how plant-based foods and supplements can control these. The proposed causative factors include insulin resistance, sex hormone imbalances, inflammation and microbial dysbiosis. There is an emerging body of work on the human gut microbiome and how it mediates feedback between the foods we eat and our bodies. The gut microbiome is also an important mediator of inflammation in the gut and systemically. A low-glycemic load diet, one rich in plant fibers and low in processed foods, has been linked to an improvement in acne, possibly through gut changes or attenuation of insulin levels. Though there is much interest in the human microbiome, there is much more unknown, especially along the gut-skin axis. Collectively, the evidence suggests that approaches such as plant-based foods and supplements may be a viable alternative to the current first line standard of care for moderate acne, which typically includes antibiotics. Though patient compliance with major dietary changes is likely much lower than with medications, it is a treatment avenue that warrants further study and development.

## 1. Introduction

Acne affects between 40 and 50 million individuals in the United States [1], including mainly adolescents and adults. Factors influencing acne development include excessive sebum production, follicular hyperkeratinization of pilosebaceous ducts, and an increased release of inflammatory mediators. Additionally, some have hypothesized that androgens and microbial colonization with *Propionibacterium acnes* contribute to the pathogenesis of acne [2,3]. The role of *P. acnes* is not clear, as this bacterium is ubiquitous. However, certain strains of acne may be more associated with acne and be pro-inflammatory [4,5,6]. Regardless of the ongoing debate regarding *P. acnes*, antibiotics used in the treatment of acne appear to have anti-inflammatory effects independent of their antimicrobial effects [7,8,9]. As a result, the first-line treatment of acne involves broad-spectrum oral and topical antibiotics, which require protracted treatments of a minimum of 3–6 months. Chronic antibiotics may have long-term side effects and detrimental effects on the host microbiome, including selection for multidrug resistant bacteria on the skin and in the gut [10]. For example, the use of clindamycin has been associated with pseudomembranous colitis [11], tetracycline has been shown to change skin color, and erythromycin can precipitate hepatic dysfunction [12]. Other medications used for acne such as isotretinoin, while effective, require close monitoring and have many side effects, including a risk of teratogenicity [13]. Therefore, there is a need for safe and effective alternatives to treat acne. Plant-based approaches have been practiced in multiple medical perspectives, including Chinese medicine and Ayurveda. Our understanding of medicinal plant efficacy and their mechanisms is growing as demand for natural, holistic approaches and fears over the ramifications of chronic antibiotic use increase. Here, we discuss the importance of the gut microbiome in acne pathogenesis and the potential for phytotherapeutic treatments (Table 1).

## 2. Methods

In January 2016, we searched Ovid MEDLINE databases for published clinical studies examining the use of oral plant-derived products for the treatment of acne vulgaris. Search terms such as “phytotherapy,” “plant medicinal product,” “herbal medicine,” “herbaceous agent,” “polyphenols,” “microbiota,” “gastrointestinal tract,” “insulin,” “diabetic” and “acne vulgaris” were used in the search strategy. Studies involving plant-derived compounds and acne vulgaris as an outcome measure were included. Bibliographies were searched for additional studies that met the inclusion criteria.

## 3. Gut Microbiome and the Skin

### 3.1. Altered Gut Function Impacts the Skin

The bacteria in our intestines function akin to an organ. Our gut bacteria perform multiple functions, including maintaining structural and functional integrity of the gut, immune system regulation, food breakdown, providing nutritional benefits to the host (biotin and vitamin K), and preventing the growth of harmful bacteria. In the 1930s, Stokes and Pillsbury used experimental evidence and anecdotes to identify an association between microbial flora and inflammation of the skin [30]. They found as many as 40% of those with acne had hypochlorhydria and hypothesized a lack of acid would induce a migration of bacteria from the colon to the small intestine and disrupt normal intestinal flora. In recent years, hypochlorhydria has been confirmed to be a significant risk factor for small intestinal bacterial overgrowth (SIBO), which can cause increased intestinal permeability (or “leaky gut”), leading to systemic inflammation [31,32]. The excess bacteria can compete with the host for nutrients, produce toxic metabolites, and cause direct injury to enterocytes in the small intestine [33]. Studies as early as 1916 suggested intestinal permeability might be augmented in acne vulgaris [34]. In one such study of 57 acne patients, researchers used a blood serum complement fixation test to demonstrate enhanced reactivity to stool-isolated coliforms in 66% of the acne patients compared to none of the control patients [34]. Later in 1983, a study involving 80 acne patients showed the presence of lipopolysaccharide (LPS) endotoxins from *Escherichia coli* in the serum of acne patients [35]. These results suggest that gut microbes may enhance the presence of circulating endotoxins in the blood of acne vulgaris patients compared to healthy controls. Although the mechanisms for how the gut and skin communicate are poorly understood, acne appears to have a potential gut-skin connection that may be a manifestation of a systemic problem involving intestinal bacteria and increased permeability.

### 3.2. Gut Microbiome Dysbiosis and Acne

The human intestine is colonized by a complex microbial ecosystem that is hypothesized to be involved in the bioavailability of orally-administered drugs, as well as a number of disease states [36]. The intestinal microbiota is a complex and dynamic bacterial community that plays an important role in human health. Alterations in microbiota composition and function have been related to different intestinal and extra-intestinal diseases [37]. The first attempts to examine the intestinal bacterial flora in acne patients was conducted in 1955 by Loveman et al. [38]. The authors concluded there were no major differences in a small subset of pathogenic bacteria. However, *Bacteroides* species were more commonly isolated from the acne patients [38]. Only a few researchers have yet investigated the intestinal microbiome in acne patients. Russian investigators who studied 114 patients with acne vulgaris noted that 54% of acne patients have differences in their intestinal flora. Additionally, they found when acne patients with dysbiosis in their intestinal flora received probiotics, there was a reduction in the duration of treatment [39]. The potential dysbiosis in the enteric microbial profile of acne patients needs further investigation and remains a potential source for alternative treatments.

Differences in the gut microflora are not unique to patients with acne vulgaris. Investigators have identified lower counts of *Bifidobacterium* in fecal specimens from patients with atopic dermatitis compared to healthy controls [40]. Furthermore, the composition and diversity of the gut microbiota in young children who develop atopic dermatitis were found to be different from children who never develop the disease [41]. The mechanisms by which the gut microbiome exerts its effects and links between the gut flora and the pathogenesis of skin disease are not clear yet and remain an active area in research.

## 4. Probiotics Improve Acne

Numerous studies have reported beneficial interactions between the human body and its microbiota. These relationships have suggested that modulation of the microbiota through prebiotics and probiotics may prevent or resolve various diseases such as pediatric infectious diseases [42], skin disease, gastrointestinal disorders [43], and allergic diseases [44]. Probiotics are live microorganisms that can alter gut homeostasis and immunity [45]. Here, we discuss current evidence supporting probiotics for the treatment of acne vulgaris.

*Bifidobacteria* and *Lactobacilli* are lactic acid-producing bacteria normally found in the gut that may assist in the treatment of inflammatory skin diseases, such as acne [46]. Physicians, as early as the 1930s, used orally-administered *Lactobacillus acidophilus* cultures as a probiotic to treat acne [47]. Despite various anecdotal reports, there was little research to determine efficacy at the time. The first formal case reports describing the use and benefits of *Lactobacilli* were not until 1961 [48]. The study gave probiotic tablets containing both *L. acidophilus* and *Lactobacillus bulgaricus* to 300 patients for 16 days with an interim two-week washout after the first eight days. The author reported 80% of patients with acne had some degree of clinical improvement, with the greatest improvement in those with severe inflammatory acne. Unfortunately the study did not have controls, and the authors simply concluded that there is an interaction between the skin manifestation of acne vulgaris and metabolic processes in the intestinal tract [48].

In recent decades, only a few studies have investigated oral probiotics in the treatment of acne vulgaris. One study tested an oral supplement composed of lyophilized *L. acidophilus* and *Bifidobacterium bifidum* in 40 patients as an adjuvant to standard antibiotics in half of the group. The authors reported patients treated with a probiotic had improved clinical outcomes and reported fewer side effects from the standard antibiotics [49]. Likewise, a Russian study tested the effectiveness of probiotics as adjuvants to standard acne treatment and found that patients taking probiotics experienced improvements sooner in their acne treatment compared to controls [39].

While the mechanism of probiotics is not well understood, recent research has shown that they may reduce oxidative stress and inflammation. Patients with acne have a high local burden of lipid peroxidation placing a high demand on blood-derived antioxidants [50]. Orally-consumed pre- and pro-biotics have been shown to reduce systemic markers of inflammation and oxidative stress [51]. Additionally, oral probiotics have been shown to regulate the release of inflammatory cytokines in the skin and reduce interleukin-1 α [46,52]. Lastly, probiotics can change the microbial community at distant sites outside of the gastrointestinal tract [53]. Therefore, the ability of oral probiotics to reduce systemic oxidative stress, regulate cytokines, and reduce inflammatory markers may all contribute to its effects on acne. Taken together, these studies suggest that the gut microbiome may play an important role in acne pathogenesis and that we can modulate it for clinical improvements, but further investigation into the mechanisms and effects of oral probiotics in acne vulgaris is needed.

## 5. Edible Plants and Acne

The beneficial role of fruits and vegetables in health maintenance is well known, though the mechanisms have only been elucidated in recent years. The gut microbiome plays an integral role in almost every aspect of human health through transformation of food and through direct signaling. Most research on dietary effects do not consider whether the effect on the host or the effect on the host’s microbiome is primarily affecting the observed response.

One of the first dietary intervention studies on acne vulgaris was performed in 2007 by Smith et al. and compared the effect of a low-glycemic load diet on acne severity [54]. Forty-three males aged 15–25 with moderate acne were fed a low-glycemic load diet for 12 weeks. The number of acne lesions, sex hormone levels, and insulin markers were compared at baseline and after intervention. The patients both lost weight and showed improvement of acne compared to a conventional Western (high-glycemic load) diet. Free androgen and fasting insulin levels were significantly lower in patients on the low-glycemic load diet. The patients designed their own diets, based on nutritional counseling, which instructed the experimental group to consume more protein and lower glycemic index carbohydrates, such as whole grains and fruits. The evidence suggests that high-glycemic load diets can contribute to acne by inducing hyperinsulinemia, while low-glycemic load diets may prevent hyperinsulinemia by lowering postprandial insulin.

In 2016, Çerman et al. again probed the relationship between glycemic load and acne, collecting self-reported food logs from 86 patients (50 with acne and 36 without) over seven days. The study included male and female patients with mild to severe acne and tracked adiponectin in addition to insulin/insulin resistance markers. Adiponectin is a protein involved in the regulation of glucose and fatty acid breakdown. Both the presence and the severity of acne positively correlated with glycemic load, but not with insulin or insulin resistance markers. Adiponectin levels were lower in acne patients than in controls, though not significantly different by severity. The glycemic load disparity between experimental and control groups was less than that in Smith’s interventional study (16 versus 73), which may explain the differences in insulin markers and reflect the normal dietary differences between young adults in Turkey and in Australia. A low-glycemic load diet balances carbohydrate intake with dietary fiber, slowing digestion and the release of sugar into the bloodstream. The recommended daily allowance (RDA) of dietary fiber is 25 g, based on a 2000 kilocalorie diet. Dietary fiber intake from 2001–2010 was 16.1 g/day for adults over age 19 [55]. This deficient consumption of dietary fiber reflects the whole grains, vegetables and fruits that average Americans are lacking on a daily basis. Though the mechanism by which this diet improves acne is unknown, complex carbohydrates, like resistant starch, insoluble fiber, and fructooligosaccharides, have been correlated with greater insulin sensitivity and less inflammation [56,57,58,59,60]. In addition to prebiotic polysaccharides, plant-based foods are also sources of bioactive polyphenols, which we discuss later.

### 5.1. Insulin

Insulin is a peptide hormone made by the pancreas that regulates carbohydrate metabolism through its influence on glucose. Evidence from multiple studies, including Smith et al., suggest insulin and carbohydrate metabolism may have a role in the etiology and severity of acne [54]. The occurrence of acne as part of various syndromes associated with insulin resistance further supports the association between insulin and acne. For example, 70% of polycystic ovary syndrome (PCOS) cases have acne symptoms. PCOS is characterized by hyperandrogenism, anovulation, polycystic ovaries, insulin resistance and hyperinsulinemia. Emiroglu et al. investigated the relationship between acne and insulin resistance in males with acne. All 22 subjects with resistant acne had impaired metabolic profiles and decreased insulin sensitivity [61].

The mechanism linking high insulin levels and acne may be through the altered proliferation of keratinocytes in the pilosebaceous unit. Hyperinsulinemia increases serum levels of insulin-like growth factor-1 and reduces serum levels of insulin-like growth factor binding protein-3 [62]. Both of these factors have been shown to increase keratinocyte proliferation and stimulate hormone production, which may contribute to the pathogenic factors of acne [61]. The gut microbiota may also contribute to insulin resistance. A Danish study of 277 non-diabetic individuals found increased populations of specific gut microbes (*Prevotella copri* and *Bacteroides vulgatus*) and an association with insulin resistance [63]. Insulin resistance and the gut may represent a new target for therapy in acne patients.

There are many plant-based foods that can improve insulin sensitivity, thereby reducing overproduction and stabilizing blood sugar. Many of the compounds responsible appear to be polyphenols, though the molecular mechanism of action is generally not understood and may vary depending on the molecule. In vitro, berry extract exposure reduced glucose uptake by human intestinal epithelial cells [20]. Foods/supplements that exert a positive effect on insulin sensitivity include olive leaf, berries (with the most data on strawberries), grapes and red wine, cinnamon, and green tea [20]. Green tea extract supplementation has been shown to decrease the number of acne lesions in postpubescent females with a trending decrease in fasting blood sugar (*p* = 0.10) and a significant decrease in total triglycerides [21]. It is important to note that there are few studies on most of these foods and their effects on glucose metabolism; therefore, results should be cautiously accepted until more, larger clinical trials are performed.

Plants from the family *Berberidaceae* are commonly used in traditional Chinese medicine for a variety of ailments, including the chronic skin conditions eczema and psoriasis [15]. This family includes the genus *Mahonia* and *Berberis*, which produce flowers and edible berries. Among many bioactive compounds in these plants, berberine is one of the most well studied. It has been shown to relieve insulin resistance in hepatic cells in vitro and to be anti-inflammatory [17,64]. Berberine and other components are antimicrobial against common skin microbes, like *Propionibacterium* acnes, *Staphylococcus* spp. and *Malassezia* spp. [16]. One of the reasons it may be effective in treating eczema and psoriasis is an antiproliferative effect on keratinocytes, which may also attenuate acne lesion development [18]. Additionally, in hamsters, berberine appeared to decrease lipogenesis by sebaceous glands, which may translate to human sebaceous glands [19]. Berberine showed strong activity against clinical isolates of *Propionibacterium acnes* isolated from acne patients. A Chinese study using Gong Lao Qu Huo herbal supplements comprised of *Mahonia* fruits was used to treat 92 patients with acne vulgaris. Ninety eight percent of the treatment group on berberine improved compared to 91% of the control group taking minocycline. Statistical analysis suggested there was no difference between the berberine and minocycline groups. This suggests that herbal supplementation may be just as effective as the standard antibiotics without the drawbacks [15].

Fruits from the genus *Garcinia* are best known for their antibacterial and weight loss effects. Weight loss may be due to leptin-like activity and the resultant decreases in insulin and insulin sensitivity, in addition to appetite suppression [22]. In sucrose-loaded mice fed *Garcinia cambogia* rind extract, there was a significant decrease in serum insulin levels (3.52 versus 1.83 ng/mL) compared to controls [22]. Male rats consuming a high fat diet showed increased serum leptin levels and decreased glucose intolerance when fed *Garcinia cambogia* ethanolic extract [65]. α and γ mangostin and phenolic ethers in *Garcinia mangostana* improved insulin sensitivity and attenuated lipopolysaccharide (LPS)-induced inflammation in vitro [66,67]. *Garcinia mangostana* extract led to an increase in the insulin-producing pancreatic β cells in normal and diabetic rats [67,68]. The loss of β cell number and function is associated with type I and II diabetes, and increasing the population has been hypothesized as a cure [69]. Although topically applied α mangostin clinically improved acne severity and inhibited growth of both *Staphylococcus epidermidis* and *P. acnes* in vitro [23,70,71], orally-administered extracts have not been tested as an acne treatment. The various activities suggest that *Garcinia* fruits may be worth studying for their effects on acne with oral supplementation.

A prebiotic supplement containing inulin, β-glucan, and blueberry polyphenols led to significantly improved glucose tolerance in adult humans, though no statistically-significant difference was observed in insulin sensitivity [72]. In diet-induced obese rats, a combination probiotic containing *Bifidobacterium*, *Lactobacillus*, *Lactococcus* and *Propionibacterium* strains improved insulin sensitivity and decreased body mass [73].

Turmeric, the ground dried root of the *Curcuma longa* plant (*Zingiberaceae* family), is known for its prominent role in curries and traditional medical systems like Chinese medicine and Ayurveda. It has shown promising results as an antimicrobial, anti-inflammatory, and antidiabetic, all activities that may improve acne vulgaris. It has been suggested that turmeric can help prevent the onset of diabetes and stabilize blood sugar [74,75,76]. Several studies in mice have shown that curcumin supplementation results in reduced glucose intolerance, hypoinsulinemia, and hyperglycemia [75]. The growth of the common skin bacteria *Staphylococcus epidermidis* and *Staphylococcus aureus* is inhibited by curcumin, which also acts synergistically with several antibiotics [28,29]. When curcumin was photoactivated, it was also able to inhibit the growth of *Propionibacterium acnes*, though unactivated curcumin did not inhibit growth [27].

### 5.2. Sex Hormones

Sex hormones, including androgens and progestins, have been implicated in acne pathogenesis. Progesterone, which peaks before menstruation and is elevated throughout gestation, has been correlated with flares of acne, psoriasis, rosacea, herpes lesions, and both atopic and allergic dermatitis [77]. However, progesterone also inhibits the enzyme 5α-reductase that transforms testosterone into 5α-dihydrotestosterone (5αDHT), a hormone that has been shown to increase proliferation of sebocytes in ex vivo sebaceous glands to a greater degree than testosterone [78]. High levels of 5αDHT have also been correlated with acne vulgaris [79]. The effect of sex hormones on acne pathology is likely more complex than absolute levels of particular hormones and could result from an imbalance between several or from the activity of 5α-reductase.

Female to male transsexual patients have in some cases suffered severe chronic acne after beginning testosterone supplementation [80]. Typical treatments like doxycycline and topical retinoids did not show an improvement, but oral isotretinoin led to clearance followed by a delayed recurrence of severe acne in both patients [80]. Another study with a larger sample size (*n* = 70) showed that acne presence and severity did increase over the first six months on testosterone, but that this condition was temporary and that only ~6% of patients had acne after long-term supplementation [81]. One explanation for the development of acne in this population is an overall increase in sebum production. Giltay and Gooren studied sebum production and hair growth in both female and male transsexual patients, where testosterone supplementation increased overall sebum production and estrogen supplementation decreased sebum production [82]. Several other studies show that women with acne have elevated levels of free testosterone and total testosterone, though this same relationship is not seen in men [83]. Estrogen can counter androgens through negative feedback loops, suggesting that increasing dietary phytoestrogens may be a better solution than attempting to decrease testosterone, which can have negative effects on male fertility since testosterone is necessary for spermatogenesis [84,85,86]. Estrogen seems to have a beneficial effect on skin, decreasing sebaceous gland size, sebum production and acne [87,88].

Phytoestrogens are present in a variety of edible plants and are famously high in soy products in the form of isoflavones [89,90]. Plants from the genus *Vitex* have been used to treat premenstrual acne, menopause symptoms and polycystic ovary syndrome. There are several polyphenols in *Vitex agnus-castus* (chasteberry) fruit, which were able to strongly bind to estrogen receptors in human breast cancer cells in vitro and are likely responsible for their clinical responses/usage [91]. The whole fruit extract of *Vitex agnus-castus* is thought to act on follicle-stimulating hormone and luteinizing hormone levels in the pituitary to increase progesterone levels [92]. *Vitex* supplementation appears to cause an increase in estrogen levels, as well [93,94]. In ovariectomized mice, *Vitex* supplementation attenuated learning and memory loss associated with low levels of estrogen, even causing an increase in estrogen receptor mRNAs [95]. Along with *Vitex agnus-castus*, hops and red clover were shown to bind to estrogen receptors in human breast cancer cells. Ginseng and licorice root showed some downstream estrogenic activity, though they did not bind to estrogen receptors [91]. These plants have been commonly used in traditional Chinese medicine for menopausal symptoms, but their role as estrogen analogues also make them promising for attenuation of acne.

### 5.3. Antimicrobial

It is accepted that acne has some microbial etiology, though the exact pathology is not known. Typical treatments, like benzoyl peroxide and antibiotics, target this component of the condition. However, there are several plant-based antimicrobials that could be viable alternatives, especially in combination with other dietary changes that address insulin resistance and hyperandrogenism. A randomized, double-blind, placebo-controlled clinical trial in India with fifty-three patients between 14 and 28 years old tested Ayurvedic plant extracts for safety and efficacy. Study subjects had mild to moderately severe acne exhibiting a minimum of 10 inflammatory lesions (papules and pustules) and five non-inflammatory lesions (blackheads). Plant extract tablets containing a mixture of *Aloe barbadensis*, *Azadirachta indica*, *Curcuma longa*, *Hemidesmus indicus*, *Terminalia chebula*, *Terminalia arjuna* and *Withania somnifera* were formulated. The study found the combined treatment of tablets and topical formulation of the plant extracts showed better results than the tablets alone, but the oral preparation was more efficacious than the topical alone [14].

The Ayurvedic formulation was also evaluated for in vitro antibacterial and anti-inflammatory activity. *Azadirachta indica* (also known as neem) contains many essential oils that have antipyretic and anthelmintic properties [96]. Additionally, it was shown to help control biliary secretion. Some Indian foods including *Curcuma longa* (turmeric) and *Azadirachta indica* have been shown to have anti-inflammatory effects by suppressing *Propionibacterium acnes*-induced reactive oxygen species and pro-inflammatory cytokines [26]. This direct anti-inflammatory property is considered to be the basis for the clinical effect of these plants in treating acne. Sunder Vati is an Ayurvedic herbal formulation containing various herbs such as *Holarrhena antidysenterica*, *Emblica officinalis*, and *Zingiber officinale.* A double-blind placebo-controlled trial of oral Sunder Vati suggested that the formulation is efficacious for the treatment of acne. Subjects treated with oral Sunder Vati had a 60% reduction in their lesion count (*p* < 0.01) [26].

Gugulipid is made from the sap of the *Commiphora mukul* tree, which is native to India. Gugulipid has been traditionally used alone or combined with other herbs for the treatment of a variety of ailments, including rheumatism, arthritis, skin diseases, and obesity. A randomized study treated twenty patients with nodulocystic acne with either tetracycline 500 mg or gugulipid 25 mg for three months. The results of the study demonstrated that both groups had a reduction in acne lesions (65.2% tetracycline vs. 68% gugulipid) (*p* > 0.05). Interestingly, patients with oily faces responded remarkably better to gugulipid [24]. This study suggests that gugulipid extract may replace tetracycline in the treatment of acne given its equivalent efficacy, improved safety profile and lack of antibiotic resistance. Gugulipid is a potent hypolipidemic agent. Apart from its hypolipidemic activity, a large number of therapeutic activities like antimicrobial, anthelmintic, anti-inflammatory, anti-arthritic, and antioxidant have been reported [25].

## 6. Conclusions

**Figure 1:**
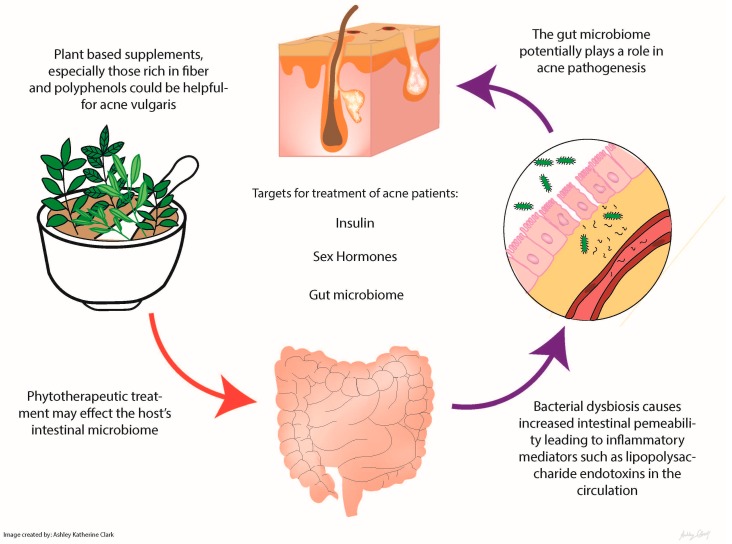
Edible plants and their influence on the gut microbiome and acne.

Acne is a multifactorial condition, but one that has been treated successfully through dietary interventions. Whether these plant-based foods primarily affect the microbiome or the human directly is unclear. It is important to note that many of these plant-based foods and spices may affect more than one factor in acne pathogenesis (e.g., insulin resistance, microbiome modulation, sex hormone balance), so without targeted evaluations, it is difficult to say which mechanism was most clinically relevant (Figure 1). In addition to the particular effects of specific plant foods, a diet high in plant matter and low in simple carbohydrates should cause a significant improvement of acne vulgaris through a variety of mechanisms. Some have suggested that acne is a visible manifestation of a systemic problem, for example insulin resistance, inflammation, gut dysbiosis, and poor nutrition. Plant-based foods and supplements, especially those rich in fiber and polyphenols, could provide a natural, low-risk intervention for acne vulgaris.

## Figures and Tables

**Table 1:** Summary of oral plant extracts for acne treatment.

Phytotherapeutic	Mechanisms	Study Type	Comparison	Number of Subjects	Outcomes	Reference
**Ayurvedic plant extracts:** ***Aloe barbadensis*, *Azadirachta indica*, *Curcuma longa*, *Hemidesmus indicus*, *Terminalia chebula*, *Terminalia arjuna* and *Withania somnifera***	Antibacterial and anti-inflammatory activity *Azadirachta indica* = antipyretic and anthelmintic *Curcuma longa* (turmeric) and *Azadirachta indica* = anti-inflammatory effects by suppressing *Propionibacterium acnes*-induced reactive oxygen species and pro-inflammatory cytokines	In vitro Double-blind, placebo-controlled clinical trial in India	Placebo	53 mild to moderately severe acne patients 14–28 years old tested	The study found that the combined treatment of tablets and topical formulation of the plant extracts showed better results than the tablets alone, but the oral preparation was more efficacious than the topical alone	Lalla et al. (2001) [14]
**Berberine**	Relieve insulin resistance Antimicrobial against common skin microbes, like *Propionibacterium* acnes, *Staphylococcus* spp. and *Malassezia* spp. Antiproliferative effect on keratinocytes Decrease lipogenesis of sebaceous glands	Randomized controlled trial Hepatic cells in vitro In hamsters	Herbal supplement vs. control group taking minocycline	92 patients with acne vulgaris	No difference between the berberine and minocycline group; this suggests that herbal supplementation may be just as effective as the standard antibiotics without the drawbacks [15]	He et al. (2015) [15]; Slobodníková et al. (2004) [16]; Hu et al. (2016) [17]; Muller et al. (1995) [18]; Seki et al. (1993) [19]
**Berry extract** (most data on strawberries)	Polyphenol with unknown molecular mechanism	In vitro	None	None	Berry extract reduced glucose uptake by human intestinal epithelial cells	Kim et al. (2016) [20] Lu et al. (2016) [21]
**Garcinia (α mangostin)**	Antibacterial, attenuation of de novo lipid synthesis	In vitro Human	None	None	Improved insulin sensitivity and attenuated LPS-induced inflammation Topical application improved acne severity	Hayamizu et al. (2003) [22]; Pan-In et al. (2015) [23]
**Green tea extract**	Epigallocatechin-3-gallate (EGCG), the major polyphenol in green tea, has potent anticarcinogenic, anti-inflammatory and antimicrobial activities; EGCG can modulate several key pathological factors of acne, including hyperseborrhea, lipogenesis, inflammation and *P*. *acnes* overgrowth	Randomized, double-blind, placebo controlled clinical trial	1500 mg of decaffeinated green tea extract vs. placebo (cellulose)	80 25–45 year-old women with post-adolescent acne	Decreased acne lesions in postpubescent females with a trending decrease in fasting blood sugar	Lu et al. (2016) [21]
**Gugulipid**	Potent hypolipidemic agent; Antimicrobial, anthelmintic, anti-inflammatory, anti-arthritic and antioxidant properties	Randomized controlled trial	Tetracycline 500 mg vs. gugulipid 25 mg	Twenty patients with nodulocystic acne	Both produced a progressive reduction in the lesions; with tetracycline, the percentage reduction in the inflammatory lesions was 65.2% as compared to 68% with gugulipid (*p* > 0.05)	Thappa et al. (1994) [24]; Goyal et al. (2011) [25]
**Sunder Vati *(Holarrhena antidysenterica*, *Emblica officinalis* and *Zingiber officinale)***	Unknown	An Indian double-blind placebo-controlled trial	Placebo	20	Treatment was associated with significant reduction (*p* < 0.01) in lesion count of approximately 60%; there was a significant reduction in the total number of inflammatory lesions within 2 weeks with further reduction at each observation period during the 6-week treatment	Paranjpe et al. (1995) [26]
**Turmeric**	Antimicrobial, anti-inflammatory and antidiabetic	In vitro	None	None	Several studies have shown growth inhibition of the common skin bacteria *Propionibacterium acnes*, *Staphylococcus epidermidis* and *Staphylococcus aureus* when curcumin is used topically	Liu et al (2013) [27]; Mun et al. (2013) [28]; Hegge et al. (2012) [29]
**Vitex**	Binds to estrogen receptors Acts on follicle-stimulating hormone and luteinizing hormone levels in the pituitary to increase progesterone levels Increase in estrogen level	In vitro	None	None	Commonly used in traditional Chinese medicine for menopausal symptoms, but their role as estrogen analogues also makes them promising for attenuation of acne	Allahtavakoli et al. (2015); Bedi et al. (2002); Ahangarpour et al. (2016)
